# Impact of COVID-19 Related Lockdown on the Frequency of Acute and Oncological Surgeries—Lessons Learned From an Austrian University Hospital

**DOI:** 10.3389/fpubh.2021.625582

**Published:** 2021-08-02

**Authors:** Julia Abram, Lukas Gasteiger, Gabriel Putzer, Patrick Spraider, Simon Mathis, Tobias Hell, Judith Martini

**Affiliations:** ^1^Department of Anaesthesiology and Intensive Care Medicine, Medical University of Innsbruck, Innsbruck, Austria; ^2^Department of Mathematics, Faculty of Mathematics, Computer Science and Physics, University of Innsbruck, Innsbruck, Austria

**Keywords:** COVID-19, elective surgery, non-elective surgery, acute surgery, public health

## Abstract

Innsbruck Medical University Hospital, Austria, provides the highest level of care for a region of approximately 1.8 million people. During the early COVID-19 outbreak in spring 2020 surgical activity was drastically reduced with the prime goal of preserving hospital capacities, especially intensive care beds. We conducted a retrospective analysis of surgical activities performed at Innsbruck Medical University Hospital during the lockdown period from March 15 to April 14, 2020 and compared these activities to the same period during the previous 5 years. Total surgical activity was reduced by 65.4% compared to the same period during the previous 5 years (*p* < 0.001); elective surgeries were reduced by 88.7%, acute surgeries by 35.3% and oncological surgeries by 47.8% compared to the previous 5 years (all *p* < 0.001). This dramatic decrease in acute and oncological surgeries can most likely be ascribed to the fact that many patients avoided health care facilities because of the strict *stay-at-home* policy and/or the fear of contracting SARS-CoV-2 in the hospital. In view of future waves, the population should be encouraged to seek medical help for acute symptoms and to attend cancer screening programs.

## Introduction

Innsbruck Medical University Hospital is the largest hospital in western Austria and provides the highest level of medical care for approximately 1.8 million people. Due to the dramatic increase in SARS-CoV-2 infections in mid-March 2020, especially in popular ski resorts in Tyrol, the authorities ordered a complete *shutdown* of daily life with public health measures such as social distancing, self-isolation and quarantine for the whole region. In accordance with these restrictions, all elective surgical procedures at Innsbruck Medical University Hospital were halted to reduce the risk of infection, harm and death from COVID-19 and to reserve personnel resources and medical equipment for the treatment of critically ill COVID-19 patients (1–3). Exceptions were only envisaged solely for acute and oncological surgery (2).

The question that inevitably arises is how these COVID-19 related restrictions affected our ability to maintain the highest quality care for all our patients, not only SARS-CoV-2- positive patients, as a large number of surgeries were cancelled or postponed that would otherwise have been treated within a tight timeframe. The aim of this retrospective analysis was to determine the effects of the lockdown period from March 15 to April 14, 2020 on the performance of surgical procedures at Innsbruck Medical University Hospital and to compare the numbers with those of the same observation period during the previous 5 years. In view of future lockdowns, our results could serve as a decision guidance for health care authorities striving to establish a safe hospital environment, where not only COVID-19 patients are assured the best possible treatment, but where all other health issues are also attended to in a timely manner.

## Materials and Methods

### Data Collection and Statistical Analysis

After receiving ethics approval (EK Nr: 1124/2020, dated 2020_05_17) from the local Ethics Committee, data on all surgical interventions performed from March 15 to April 14 in the years 2015 to 2020 (*n* = 21.481) were extracted from the surgical planning software myMedis (Getinge, IT Solutions GmbH, Sweden). In a second step all surgical interventions were classified according to the organizational unit, date and time of surgery (core time: 7 a.m.−5 p.m., shift time: 5 p.m.−7 a.m.; weekday versus weekend); in a second step all surgical interventions were manually classified as elective, acute or oncology-related interventions by two medical doctors. The following interventions were classified as oncology-related surgeries: invasive diagnostics requiring anesthesia, major- and minor primary oncological surgery and follow-up procedures. Additionally, all patients were classified according to age, gender and American Society of Anaesthesiologists (ASA) physical status classification system.

Statistical analyses were conducted using R, version 3.5.1. All statistical assessments were two-sided and a significance level of 5% was used. We present the absolute number of surgeries with a 95% CI (confidence interval) for the period 2015 to 2019, other categorical variables as frequencies (%) and continuous data as mean (95% CI). We applied the Exact Poisson test to assess the difference in the number of surgeries between 2015–2019 and 2020, Fisher's exact test for binary variables and the Welch two sample *t*-test for continuous variables. We show effect size with estimated median differences for continuous data and odds ratios (OR) for binary variables, with 95% CIs.

## Results

### Patient Demographics, Comorbidities, and Country of Origin

Age distribution of surgical patients was similar during the compared periods, whereas female gender was significantly more frequent in 2020 (54.3 vs. 51.2%; *p* = 0.0305; Table 1). Additionally, during the 2020 lockdown period significantly fewer surgical patients were categorized as ASA 1 (21.7 vs. 31.6%; *p* < 0.0001; Table 1), whereas significantly more patients were categorized as ASA 3 and ASA 4 (ASA 3: 34.4 vs. 24.1%; *p* < 0.0001; ASA 4: 5.3 vs. 2.7%; *p* < 0.0001; Table 1).

**Table 1:** Presented are age, gender, comorbidities, and country of origin of patients during the observation period in 2020 compared to the same period in 2015-19.

	**Mean for 2015-2019 with 95% CI** ^**a**^ **(***n*** = 20,090)**	**2020 (*n* = 1,391)**	**Estimate with 95% CI** ^**b**^	**Decrease (%)** ^**c**^	***p*** **-value** ^**d**^	**Missing**
Age (years)	54.4 (54.1 to 54.8)	54.8	−0.3 (−1.5 to 0.8)	−0.6 (−1.2 to 0)	0.5816	0/0
Gender (female)	51.2% (50.2% to 52.3%)	54.3%	1.1 (1 to 1.3)	−5.9 (−8.2 to −3.8)	0.0305	0/0
Country of origin (Tyrol)	89.7% (88.6% to 90.7%)	94.2%	1.9 (1.5 to 2.4)	−5.1 (−6.3 to −3.9)	<0.0001	0/0
ASA Score = 1	31.6% (28.3% to 34.9%)	21.7%	0.6 (0.5 to 0.7)	31.3 (23.3 to 37.8)	<0.0001	345/6736
ASA Score = 2	41.5% (38.2% to 44.8%)	38%	0.9 (0.8 to 1)	8.5 (0.7 to 15.2)	0.0224	345/6736
ASA Score = 3	24.1% (23.6% to 24.5%)	34.4%	1.7 (1.4 to 1.9)	−43 (−45.6 to −40.5)	<0.0001	345/6736
ASA Score = 4	2.7% (2.2% to 3.1%)	5.3%	2 (1.5 to 2.7)	−98.2 (−138.6 to −69.5)	<0.0001	345/6736
ASA Score = 5	0.2% (0.1% to 0.3%)	0.6%	3.2 (1.1 to 8.1)	−224.7 (−815.7 to −97.4)	0.0191	345/6736

*ASA refers to the American Society of Anaesthesiologists physical status classification system*.

a *Assessed by t-test*.

b *Odds ratios assessed by Fisher's Exact Test for binary variables and estimated mean difference assessed by Welch two sample t-test for continuous variables*.

c Calculated as 100/(mean 2015-2019)*(mean 2015-2019-2020).

d *Assessed by Fisher's Exact Test for categorical variables and Welch two sample t-test for continuous variables*.

During the 2020 lockdown period patients undergoing surgery were more frequently inhabitants of Tyrol than in previous years (94.2 vs. 89.7%; *p* < 0.0001; Table 1).

### Numbers of Surgical Interventions

Surgical activities at 13 surgical departments were analyzed (Table 2). Between March 15 and April 14, 2020 1.391 surgical interventions were performed at Innsbruck Medical University Hospital, which is a decrease of 65.4% as compared to the mean number of surgeries performed during the same time frame in the previous 5 years (2015–2019; mean = 4.018; *p* < 0.0001; Table 2). Of these interventions, 244 were elective (−88.7%; *p* < 0.0001), 903 acute (−35.3%; *p* < 0.0001) and 241 oncological interventions (−47.8%; *p* < 0.0001). The largest reduction in surgical activity was seen at the Department of Orthopaedic Surgery (−84.5%; Table 2); the smallest reduction at the Department of Gynaecology and Obstetrics (−28.9%; Table 2).

**Table 2:** Presented are the total number of surgical procedures and the procedures in each surgical subspecialty during the observation period in 2020 compared to the same period in 2015-19.

	**Mean 2015-2019 with 95% CI** ^**a**^	**2020**	**Estimate with 95% CI** ^**b**^	**Decrease (%)** ^**c**^	***p*** **-value** ^**a**^
Total number of surgeries	4018 (3962.6 to 4074)	1391	2627 (2571.6 to 2683)	65.4 (64.9 to 65.9)	<0.0001
Elective surgeries	2153.8 (2113.3 to 2194.9)	244	1909.8 (1869.3 to 1950.9)	88.7 (88.5 to 88.9)	<0.0001
Acute surgeries	1395.6 (1363 to 1428.7)	903	492.6 (460 to 525.7)	35.3 (33.8 to 36.8)	<0.0001
Oncological surgeries	461.6 (443 to 480.8)	241	220.6 (202 to 239.8)	47.8 (45.6 to 49.9)	<0.0001
Surgical subspecialities					
Ophthalmology	880.4 (854.6 to 906.8)	153	727.4 (701.6 to 753.8)	82.6 (82.1 to 83.1)	<0.0001
Vascular surgery	174.6 (163.2 to 186.6)	118	56.6 (45.2 to 68.6)	32.4 (27.7 to 36.8)	<0.0001
Cardiac surgery	164.4 (153.4 to 176)	96	68.4 (57.4 to 80)	41.6 (37.4 to 45.5)	<0.0001
Paediatric surgery	55.6 (49.3 to 62.5)	13	42.6 (36.3 to 49.5)	76.6 (73.6 to 79.2)	<0.0001
Gynaecology and obstetrics	459.8 (441.2 to 479)	327	132.8 (114.2 to 152)	28.9 (25.9 to 31.7)	<0.0001
Ear, nose and throat surgery	210.6 (198.1 to 223.7)	42	168.6 (156.1 to 181.7)	80.1 (78.8 to 81.2)	<0.0001
Cranio, maxillofacial and oral surgery	296 (281.1 to 311.5)	52	244 (229.1 to 259.5)	82.4 (81.5 to 83.3)	<0.0001
Neurosurgery	236.2 (222.9 to 250.1)	78	158.2 (144.9 to 172.1)	67 (65 to 68.8)	<0.0001
Orthopaedic surgery	233 (219.8 to 246.8)	36	197 (183.8 to 210.8)	84.5 (83.6 to 85.4)	<0.0001
Plastic surgery	272.6 (258.3 to 287.5)	83	189.6 (175.3 to 204.5)	69.6 (67.9 to 71.1)	<0.0001
Trauma surgery	376.6 (359.8 to 394)	122	254.6 (237.8 to 272)	67.6 (66.1 to 69)	<0.0001
Urology	290.4 (275.7 to 305.7)	87	203.4 (188.7 to 218.7)	70 (68.4 to 71.5)	<0.0001
Visceral, transplant and thoracic surgery	367.8 (351.2 to 385)	184	183.8 (167.2 to 201)	50 (47.6 to 52.2)	<0.0001

a *Assessed by Poisson Test*.

b *Estimated mean difference assessed by Poisson Test*.

c Calculated as 100/(mean 2015-2019)*(mean 2015-2019-2020).

### Timing of Performed Surgical Procedures

**Figure 1:**
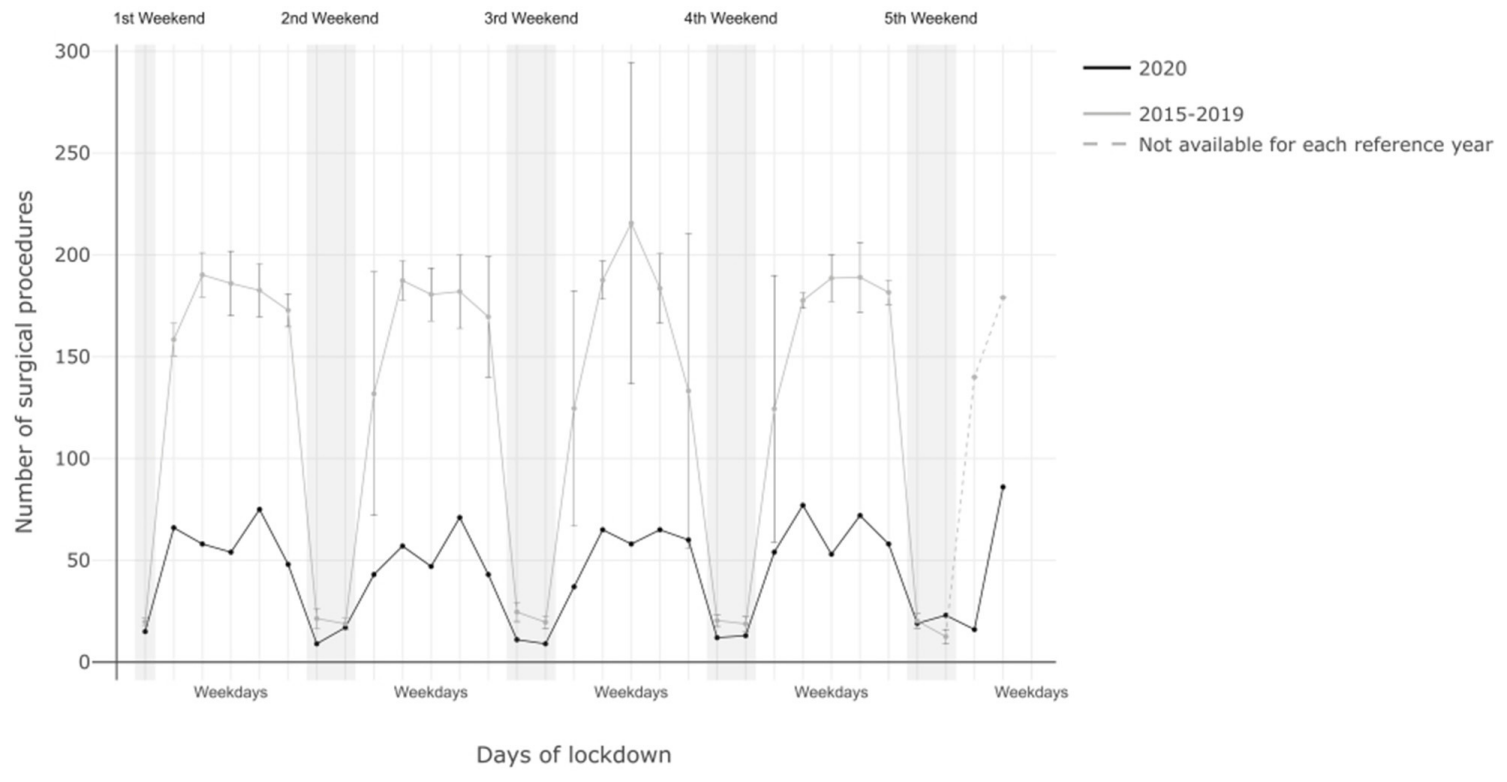
Shown is the timing of surgical procedures performed in the observation period in 2020 compared to the same period in 2015-19.

In the 2020 lockdown phase, 1.105 surgical interventions were performed during core time (7 a.m.−5 p.m.), which is a reduction of 69% as compared to the previous 5 years (*p* < 0.0001). Surgical interventions performed during night shift hours were reduced by 36.4% (*p* < 0.0001). Analysis of the weekends (Saturday and Sunday) showed, that surgical activities across all departments were significantly reduced during the 2020 lockdown as compared to the previous 5 years (−28.4%; *p* < 0.0001; Figure 1).

### Oncological Surgery

During the observed period in 2020 241 oncological surgeries were performed. Compared to the mean of the previous 5 years, this corresponds to a reduction of 47.8% (*p* < 0.0001).

**Figure 2:**
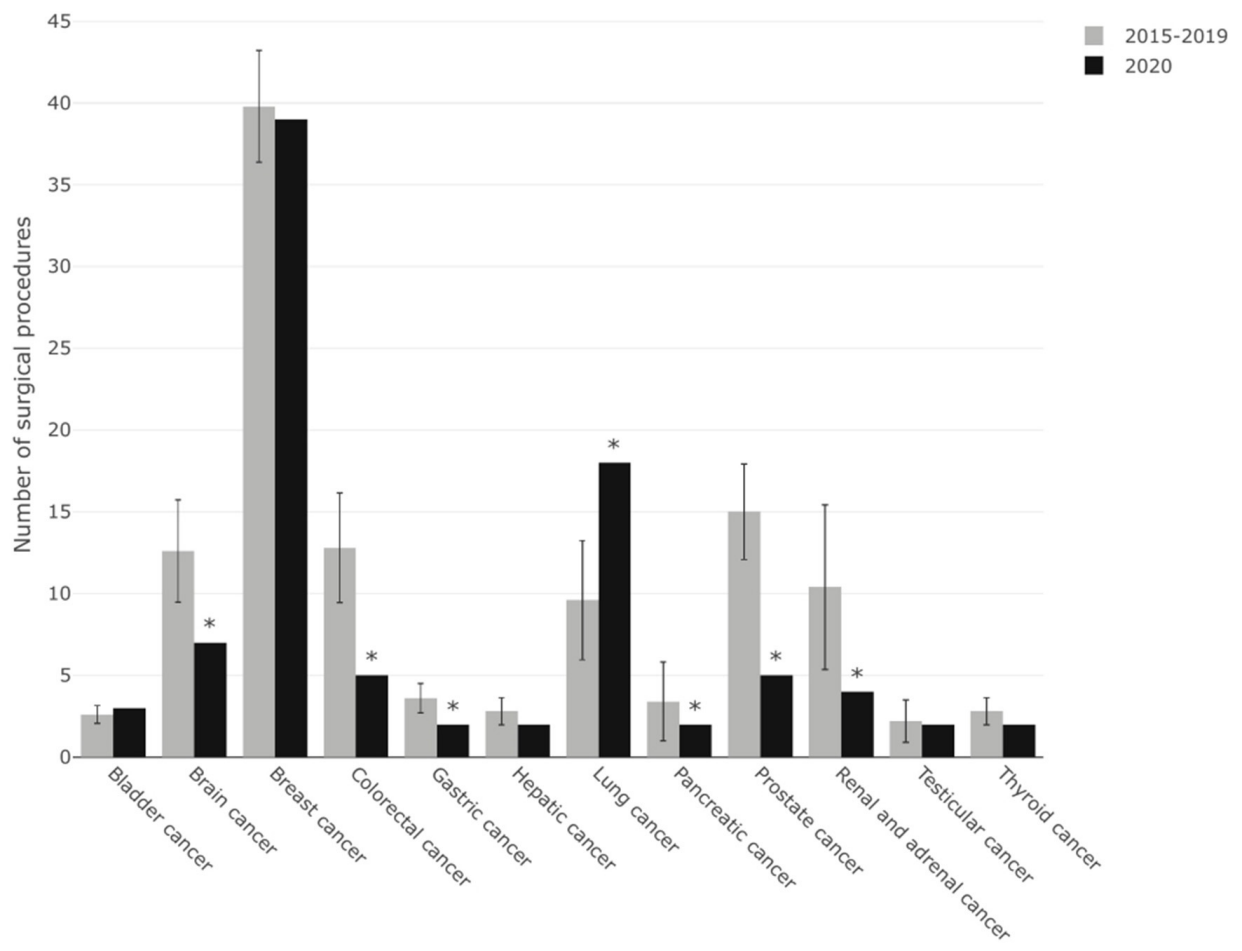
Shown is the number of oncologic surgeries for major oncologic entities performed during the 2020 observation period compared with the same period in 2015-19.

Detailed analysis of each single oncological entity, however, revealed that numbers of major surgeries for breast, thyroid, hepatic, bladder and testicular cancers were not significantly reduced during the 2020 lockdown period as compared to the previous 5 years. In contrast, a significant reduction in major oncological surgeries was seen for colorectal, pancreatic, gastric, renal, prostatic and brain tumors. Lung cancer surgery was significantly increased during the 2020 lockdown period (Figure 2).

## Discussion

Our data from the 13 surgical departments at Innsbruck Medical University Hospital show that elective surgical interventions were reduced by 88.7% during the lockdown period in 2020 as compared to the same time frame during the previous 5 years. Most interestingly, also acute and oncological surgeries were significantly reduced. Patients were generally sicker as evidenced by significantly higher ASA scores. The reduction in surgical interventions applied to all surgical disciplines and was more pronounced on weekdays than on weekends.

The reduction in trauma-associated acute surgeries (decrease of 67.6%; *p* < 0.0001) is most likely due to quarantine regulations in force at the time, which essentially prohibited all accident-prone outdoor activities such as skiing, mountaineering and paragliding. Additionally, with the closing of the Austrian border on March 15, about 150,000 tourists left the country, thereby significantly reducing the number of potential patients. Unexpected and therefore more difficult to explain, however, was the decline in acute interventions in other surgical disciplines, such as acute vascular occlusion in vascular surgery, acute appendicitis in general surgery or abscess incisions in oral and maxillofacial surgery. One possible explanation for the steep decline in acute interventions could be the so-called “COVID-19 collateral damage syndrome” (4). Many different medical specialists (surgeons, emergency physicians, cardiologists and neurologists) report that the number of acute patients has decreased during the pandemic (5–7) and that acute conditions led to more complications due to delayed presentation (8). The decline in emergency surgeries is comparable to observations reported from other countries, where a reduction of approximately 30% in acute surgical activity was described (9). One possible explanation for this uniform finding could be that lockdown measures such as the rigorous *stay-at-home* directive may have discouraged symptomatic patients from seeking early emergency treatment for non-COVID-19-related medical conditions; second, it can be hypothesized that also the fear of contracting COVID-19 in a health care facility may have further decreased the number of patients seeking medical help. This aspect is notable, especially in view of the potential collateral health damage that can occur, if certain medical conditions are not treated within a short timeframe (4). At Innsbruck Medical University Hospital the decrease in acute interventions can probably be explained by fewer patients coming to emergency departments, as there was never a shortage of staff, medical equipment or intensive care capacity. It may also be possible that the pandemic helped to accelerate implementations of recent findings into clinical practice, such as a more conservative, antibiotic based approach in case of simple uncomplicated appendicitis, which has been shown to be a safe alternative to surgery (10–12). Another important finding is that also oncological surgery was significantly reduced during the lockdown period. This is in fact surprising as scheduled or even short-dated oncologic procedures were not cancelled or postponed. This may be attributable to a COVID-19-related reduction in allocation from primary health care centers and to the fact that diagnostic procedures also significantly declined during this period. In addition, a decline in incidental findings and in positive results from cancer screening programs may have probably led to a reduction in oncological surgeries. This finding is alarming, as even modest delays in cancer surgery have a significant impact on survival (13).

Austrian mortality data show that between March 1 and April 30, 2020 overall mortality increased by 1%, peaking in mid-April, when mortality was 16% higher than in the same time period during the previous 5 years (14). However, only 3.9% of all deaths during these 2 months were causally related to COVID-19 infection, whereas death rates from cerebrovascular causes, from myocardial infarction and from lung cancer were still higher. Cerebrovascular diseases and cancer remain the leading causes of death. During the current pandemic it is therefore of paramount importance to strengthen the public's awareness for cerebrovascular diseases and its risk factors as well as the importance of attending cancer screening programs.

## Conclusions

In summary, our data show that during the lockdown period from March 15 to April 14, 2020 acute and oncology patients were less likely to undergo surgical interventions by comparison to the same period in the previous 5 years. These findings emphasize once again a major challenge of the current pandemic, namely the difficult provision of access to health care facilities and medical services to the entire population, not only persons infected with SARS-CoV-2. On the one hand, this implies efforts to keep medical systems running, including cancer screening programs and diagnostic procedures; on the other hand, massive efforts should be undertaken in healthcare facilities to reassure patients that every possible precaution is being taken to prevent viral transmission, including rigorous and repeated testing of all healthcare workers and all admitted patients. In fact, since the end of May 2020 all surgical patients at the Innsbruck Medical University Hospital have been screened for SARS-CoV2 and since December 2020 all health care workers are obligated for weekly testing. It is of prime importance to create a healthcare environment where patients feel safe and are not afraid to seek medical help. If this is not ensured, the collateral damage from non-COVID-19-related health issues will cause enormous social and economic consequences for the entire healthcare system.

## Data Availability Statement

The raw data supporting the conclusions of this article will be made available by the authors, without undue reservation.

## Ethics Statement

The study was approved by the Ethics Committee of the Medical University of Innsbruck, Austria.

## Author Contributions

LG, GP, and JM: conceptualization, methodology, validation, writing the original draft preparation, supervision, and project administration. JA, LG, GP, and JM: formal analysis. JA and TH: data curation. JA, LG, GP, PS, SM, TH, and JM: writing the review and editing. TH: visualization. All authors contributed to the article and approved the submitted version.

## Conflict of Interest

The authors declare that the research was conducted in the absence of any commercial or financial relationships that could be construed as a potential conflict of interest.

## Publisher's Note

All claims expressed in this article are solely those of the authors and do not necessarily represent those of their affiliated organizations, or those of the publisher, the editors and the reviewers. Any product that may be evaluated in this article, or claim that may be made by its manufacturer, is not guaranteed or endorsed by the publisher.
